# Testicular seminoma with a progressing pulmonary nodule and mediastinal lymphadenopathy without retroperitoneal metastasis

**DOI:** 10.1002/iju5.12191

**Published:** 2020-07-07

**Authors:** Yuta Sano, Motohiro Fujiwara, Takeshi Yuasa, Yoshinobu Komai, Tatsuya Yamamoto, Atsushi Kohno, Masayuki Nakao, Kentaro Inamura, Junji Yonese

**Affiliations:** ^1^ Department of Urology Cancer Institute Hospital Japanese Foundation for Cancer Research Tokyo Japan; ^2^ Department of Radiology Cancer Institute Hospital Japanese Foundation for Cancer Research Tokyo Japan; ^3^ Department of Thoracic Surgical Oncology Cancer Institute Hospital Japanese Foundation for Cancer Research Tokyo Japan; ^4^ Department of Pathology Cancer Institute Hospital Japanese Foundation for Cancer Research Tokyo Japan

**Keywords:** mediastinal lymph node, sarcoid‐like reaction, sarcoidosis, seminoma, testicular germ cell cancer

## Abstract

**Introduction:**

Testicular germ cell cancer has a relatively good prognosis even if visceral and/or lymph node metastases are present thanks to chemotherapy. Yet chemotherapy can lead to various adverse events. Therefore, it is crucial to distinguish whether a suspected metastatic disease is metastasis or not.

**Case presentation:**

A 33‐year‐old male visited our hospital to receive subsequent therapy for suspected recurrent seminoma with a progressing pulmonary nodule and mediastinal lymphadenopathy after orchiectomy. The pathological diagnosis of needle aspiration and resected specimen of the several lesions was consistent with epithelioid cell granuloma without caseous necrosis. Based on these findings, the lung and mediastinal lymph node lesions were diagnosed as sarcoidosis.

**Conclusion:**

In cases where the simultaneous occurrence of other benign or malignant diseases is suspected, pathological confirmation is necessary for appropriate decision‐making.

**Abbreviations & Acronyms**
^18^F‐FDG: ^18^F‐fluorodeoxyglucose
ACE: angiotensin converting enzyme
Ca: calcium
CT: computed tomography
EBUS‐TBNA: endobronchial ultrasound‐guided transbronchial needle aspiration
GCC: germ cell cancer
PET: positron emission tomography
sIL2‐R: soluble interleukin 2 receptor

**Keynote message**

We report a case of seminoma with a progressing pulmonary nodule and mediastinal lymphadenopathy without retroperitoneal metastasis. Histopathological confirmation is necessary to avoid misdiagnosis and unnecessary chemotherapy.

## Case presentation

**Fig. 1:**
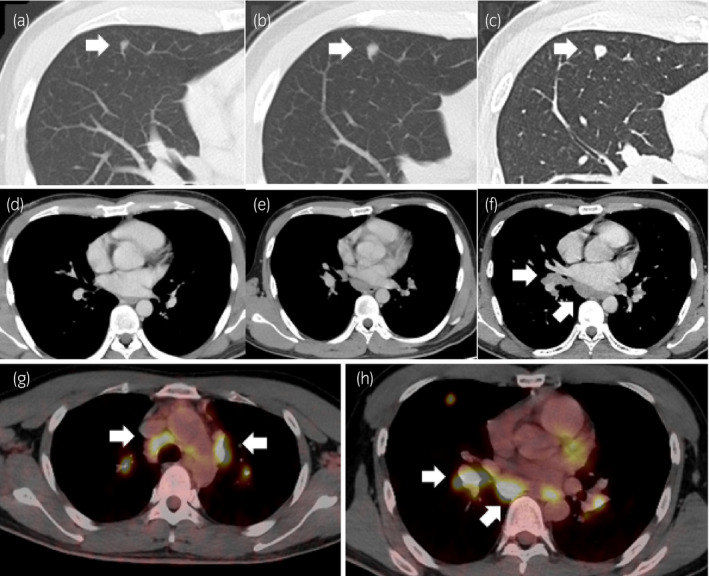
Imaging findings of a lung nodule with mediastinal lymphadenopathy of a patient with testicular seminoma after right orchiectomy. Progressing lung nodule at (a) 3, (b) 6, and (c) 9 months after orchiectomy. Development of mediastinal lymphadenopathy at (d) 3, (e) 6, and (f) 9 months after orchiectomy. PET/CT fusion images revealed accumulation of ^18^F‐labeled fluorodeoxyglucose in the lung nodule and mediastinal lymph nodes.

A patient visited our hospital in January 2020 for subsequent therapy for suspected recurrent GCC. He had a history of right orchiectomy for testicular GCC 10 months previously. He had no other past history or allergies. His pathological diagnosis at that time had been pT1 seminoma 17 mm in diameter with no rete testis invasion. At the time of his suspected recurrence, there were no apparent metastatic lesions and all tumor markers were within their normal ranges. During follow‐up surveillance, a thoraco‐abdominal CT scan revealed a progressing lung nodule with multiple enlarged mediastinal lymph nodes. The pulmonary nodules (Fig. 1a–c) and mediastinal lymph nodes (Fig. 1d–f) tended to increase in size over two months of observation. ^18^F‐FDG‐PET/CT also revealed several enlarged and hypermetabolic mediastinal nodes (Fig. 1g,h). Retroperitoneal lymph node enlargement, in contrast, was not observed. All testicular tumor markers remained negative (lactate dehydrogenase 175 U/L, alpha‐fetoprotein 2.2 ng/mL, human chorionic gonadotropin <1.0 mIU/mL). There were no ocular or skin lesions, which were suggestive for sarcoidosis. In addition, serum levels of sIL2‐R, adjusted Ca, and ACE were not elevated (sIL2‐R 479 U/mL, adjusted Ca 9.3 mg/dL, ACE 20 U/L). Through discussion among the urologists, pulmonologists, and radiologists involved in the management of the patients, we decided to proceed with pathological confirmation of the lesions because this case had characteristics that were atypical of metastatic recurrence of seminoma. We concluded that it was necessary to rule out the possibility of coexisting sarcoidosis and malignant lymphoma.

**Fig. 2:**
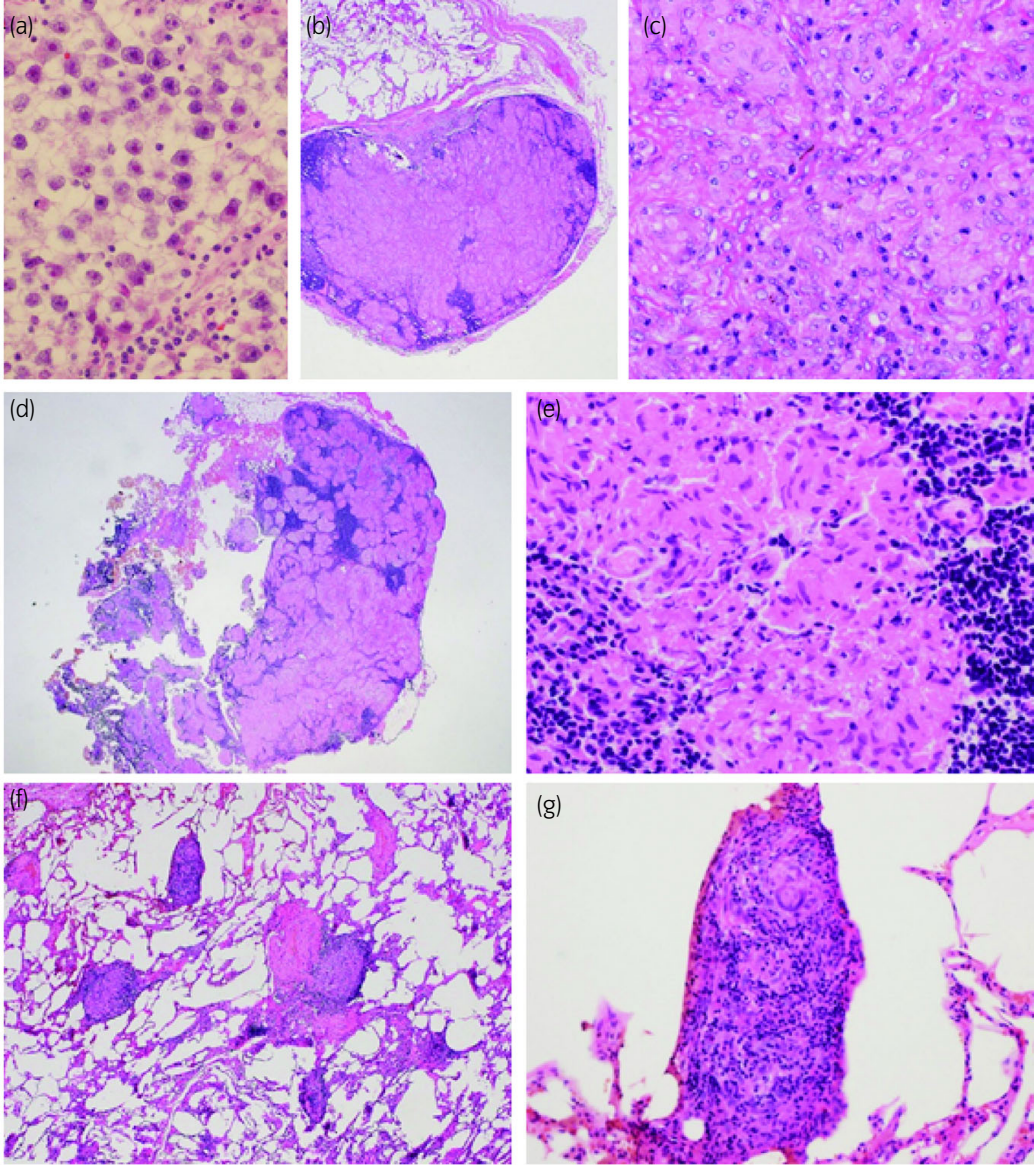
Representative histology of primary seminoma, lung nodule, and mediastinal lymph nodes. The primary lesion was diagnosed as seminoma (a: objective 40×). The lung nodule (b: objective 4×, c: objective 20×) and a mediastinal lymph node (d: objective 2×, e: objective 40×) were surgically removed and diagnosed as epithelioid cell granuloma without caseous necrosis. Small lesions of epithelioid cell granuloma without caseous necrosis were scattered around the lung nodule (e: objective 4×, f: objective 40×).

Samples of the mediastinal lymph nodes were taken by EBUS‐TBNA. The pathological diagnosis of these lesions was not seminoma but rather epithelioid cell granuloma without necrosis. No neoplastic cells originating from the primary seminoma were observed (Fig. 2a). Consequently, a thoracoscopic partial resection of the right middle lobe of the lung and an excisional biopsy of a mediastinal lymph node were performed in February 2020 for further examination. Both the lung nodule and the mediastinal lymph node showed the same pathological features as the EBUS‐TBNA materials and were diagnosed as epithelioid cell granuloma without caseous necrosis; no neoplastic cells were observed in either lesion (Fig. 2b–e). In addition, small lesions of epithelioid cell granuloma without caseous necrosis were scattered around the lung nodule (Fig. 2f,g). Based on these pathological findings, the lung, mediastinal, and hilar lymph node lesions were diagnosed as sarcoidosis. We are planning follow‐up for the sarcoidosis with attention to the possible appearance of systemic symptoms as well as continued surveillance of the stage I non‐metastatic seminoma.

## Discussion

In this case, enlargement of the mediastinal and hilar lymph nodes was observed without retroperitoneal lymphadenopathy, which is an atypical metastatic pattern for seminoma. This required us to rule out the possibility of malignant lymphoma and sarcoidosis. Testicular GCCs, especially seminoma, have a strong tendency toward lymphatic dissemination. In a report by White *et al.* on 92 cases of seminoma, there were no cases featuring intrathoracic/mediastinal metastasis without retroperitoneal lymphadenopathy.^1^ Although its precise incidence is difficult to calculate, metastatic seminoma without enlargement of retroperitoneal lymph nodes is extremely rare. Consequently, stage 1 seminoma is usually assessed through sentinel node biopsy, which is based on the assumption of a sequential dissemination of metastases through the retroperitoneal lymph nodes, and has historically been treated with prophylactic radiation therapy, though this is no longer done due to the risk of secondary malignancy.^2^, ^3^

Sarcoidosis is an unexplained multi‐organ disease characterized by noncaseating epithelioid cell granuloma. It occurs most often in young and middle‐aged patients, and often appears on chest X‐ray as bilateral hilar lymphadenopathy, but also often involves the eyes and skin.^4^ Diagnosis is based on histological evidence of granuloma without caseous necrosis in addition to clinical and radiological findings.^5^, ^6^ Most patients with sarcoidosis do not require treatment and undergo spontaneous regression without any treatment.^6^ Then, medical treatment is indicated only when symptoms are progressive, causing life‐ or organ‐threatening disease.^5^ Glucocorticoids are the mainstay of therapy even though the ideal dose and duration are not known because of the lack of data from randomized controlled studies.^5^ Methotrexate is the most commonly used corticosteroid‐sparing agent for corticosteroid‐resistant disease or corticosteroid‐intolerant patients.^5^ Sarcoidosis sometimes mimics metastatic progression in radiological findings. Moreover, there are cases like ours in which sarcoidosis and malignant diseases coexist, leading to diagnostic dilemmas.^7^, ^8^ Paparel *et al.* reviewed 64 cases in which sarcoidosis coexisted in patients with testicular cancer.^7^ Seminoma was the most common histological type of testicular cancer among these patients (63%, 40/64).^7^ The coexistence of sarcoidosis and testicular cancer did not change the management of testicular cancer or the overall prognosis.^7^

Mistaking sarcoidosis for metastatic lesions can lead to unnecessary chemotherapy and must be avoided. At the same time, mistaking metastatic disease for sarcoidosis can lead to inadequate treatment and must also be avoided. Spiekermann *et al.* reviewed 59 cases with coexisting cancer and sarcoidosis including four cases of seminoma.^8^, ^9^, ^10^, ^11^, ^12^ A few of these cases were actually simultaneous occurrences of sarcoidosis and metastases leading to insufficient treatment. In our case, because we did not subject every mediastinal lymph node to histological analysis, it remains possible that metastatic seminoma could also coexist in the residual mediastinal lymph nodes. Therefore, we intend to continue with our careful monitoring and follow‐up of our patient over the long term.

Another confounding factor known as paraneoplastic sarcoid‐like reaction should also be considered. In this phenomenon, reportedly found in 4.4% of solid tumors,^13^ the host's immune response is elicited against antigenic substances released from the tumor,^13^ resulting in non‐caseous granuloma similar to sarcoidosis. Most paraneoplastic sarcoid‐like reactions are found in primary tumors and regional lymph nodes, but some occur in distant organs and lymph nodes.^14^, ^15^ Several seminoma cases with coexisting sarcoidosis and paraneoplastic sarcoid‐like reaction have also been reported.^16^, ^17^, ^18^ Adequate pathological examination is necessary in order to confirm the absence of malignant metastasis.

In conclusion, it is important for physicians treating GCC in clinical practice to remain alert to the possibility that other benign or malignant diseases may occur simultaneously. When a patient demonstrates an unusual metastatic pattern, we should consider the possibility of coexisting sarcoidosis, especially in the intra‐thoracic region. Histopathological confirmation is necessary to ensure precise diagnosis and appropriate decision‐making and to avoid misdiagnosis and subsequent unnecessary chemotherapy.

## Conflict of interest

The authors declare no conflict of interest.

## References

[1] White PM , Adamson DJ , Howard GC , Wright AR . Imaging of the thorax in the management of germ cell testicular tumours. Clin. Radiol. 1999; 54: 207–11. 10210337 10.1016/s0009-9260(99)91152-2

[2] Blok JM , Kerst JM , Vegt E *et al* Sentinel node biopsy in clinical stage I testicular cancer enables early detection of occult metastatic disease. BJU Int. 2019; 124: 424–30. 30417511 10.1111/bju.14618 PMC6850062

[3] Aydin AM , Zemp L , Cheriyan SK , Sexton WJ , Johnstone PAS . Contemporary management of early stage testicular seminoma. Transl. Androl. Urol. 2020; 9(Suppl 1): S36–S44. 32055484 10.21037/tau.2019.09.32 PMC6995845

[4] Grunewald J , Grutters JC , Arkema EV , Saketkoo LA , Moller DR , Müller‐Quernheim J . Sarcoidosis. Nat. Rev. Dis. Primers 2019; 5: 45. 31273209 10.1038/s41572-019-0096-x

[5] Ungprasert P , Ryu JH , Matteson EL . Clinical manifestations, diagnosis, and treatment of sarcoidosis. Mayo Clin. Proc. Innov. Qual. Outcomes 2019; 2: 358–75. 10.1016/j.mayocpiqo.2019.04.006 PMC6713839 31485575

[6] Bargagli E , Prasse E . Sarcoidosis: a review for the internist. Intern. Emergency Med. 2018; 13: 325–31. 10.1007/s11739-017-1778-6 29299831

[7] Paparel P , Devonec M , Perrin P *et al* Association between sarcoidosis and testicular carcinoma: a diagnostic pitfall. Sarcoidosis Vasc. Diffuse Lung Dis. 2007; 24: 95–101. 18496978

[8] Spiekermann C , Kuhlencord M , Huss S , Rudack C , Weiss D . Coexistence of sarcoidosis and metastatic lesions: a diagnostic and therapeutic dilemma. Oncol. Lett. 2017; 14: 7643–52. 29344212 10.3892/ol.2017.7247 PMC5755156

[9] Claus F , De Wever L , Moerman P . Coincidence of seminoma and sarcoidosis in two patients presenting with peritoneal surface disease. Int. J. Urol. 2012; 19: 1126. 22906149 10.1111/j.1442-2042.2012.03129.x

[10] Teo M , McCarthy JE , Brady AP , Curran DR , Power DG . A case of sarcoidosis in a patient with testicular cancer post stem cell transplant. Acta Oncol. 2013; 52: 869–71. 22708529 10.3109/0284186X.2012.689854

[11] Tjan‐Heijnen VC , Vlasveld LT , Pernet FP , Pauwels P , De Mulder PH . Coincidence of seminoma and sarcoidosis: a myth or fact? Ann. Oncol. 1998; 9: 321–5. 9602267 10.1023/a:1008220002148

[12] Kachalia AG , Ochieng P , Kachalia K , Rahman H . Rare coexistence of sarcoidosis and lung adenocarcinoma. Respir. Med Case Rep. 2014; 12: 4–6. 26029525 10.1016/j.rmcr.2013.12.008 PMC4061445

[13] Brincker H . Sarcoid reactions in malignant tumours. Cancer Treat. Rev. 1986; 13: 147–56. 3536088 10.1016/0305-7372(86)90002-2

[14] Beutler BD , Cohen PR . Sarcoma‐associated sarcoid reaction: Report of cutaneous sarcoid reaction in a patient with liposarcoma. World J. Clin. Cases 2015; 3: 988–92. 26677448 10.12998/wjcc.v3.i12.988 PMC4677087

[15] Hunsaker A , Munden R , Pugatch R *et al* Sarcoidlike reaction in patients with malignancy. Radiology 1996; 200: 255–61. 8657922 10.1148/radiology.200.1.8657922

[16] Jegannathen A , Taylor MB , Jones M , Logue JP . Testicular seminoma with mediastinal lymphadenopathy — a diagnostic pitfall. Br. J. Radiol. 2009; 82: e85–e86. 19386953 10.1259/bjr/40671180

[17] Tanizawa K , Tanaka E , Hashimoto S *et al* Paradoxical development of a sarcoid‐like reaction during successful chemotherapy for seminoma. Intern. Med. 2010; 49: 1423–6. 20647660 10.2169/internalmedicine.49.3444

[18] Ishii H , Igata F , Nabeshima K , Kushima H , Watanabe K . Mediastinal seminoma with an elevated level of serum angiotensin‐converting enzyme. Intern. Med. 2015; 54: 1909–12. 26234235 10.2169/internalmedicine.54.3953

